# Kinetic profiling of in vivo lung cellular inflammatory responses to mechanical ventilation

**DOI:** 10.1152/ajplung.00048.2015

**Published:** 2015-03-13

**Authors:** Samantha J. Woods, Alicia A. C. Waite, Kieran P. O'Dea, Paul Halford, Masao Takata, Michael R. Wilson

**Affiliations:** Section of Anaesthetics, Pain Medicine and Intensive Care, Faculty of Medicine, Imperial College London, Chelsea and Westminster Hospital, London, United Kingdom

**Keywords:** lipopolysaccharide, mitogen-activated protein kinase, nuclear factor-κB, inflammation, ventilator-induced lung injury

## Abstract

Mechanical ventilation, through overdistension of the lung, induces substantial inflammation that is thought to increase mortality among critically ill patients. The mechanotransduction processes involved in converting lung distension into inflammation during this ventilator-induced lung injury (VILI) remain unclear, although many cell types have been shown to be involved in its pathogenesis. This study aimed to identify the profile of in vivo lung cellular activation that occurs during the initiation of VILI. This was achieved using a flow cytometry-based method to quantify the phosphorylation of several markers (p38, ERK1/2, MAPK-activated protein kinase 2, and NF-κB) of inflammatory pathway activation within individual cell types. Anesthetized C57BL/6 mice were ventilated with low (7 ml/kg), intermediate (30 ml/kg), or high (40 ml/kg) tidal volumes for 1, 5, or 15 min followed by immediate fixing and processing of the lungs. Surprisingly, the pulmonary endothelium was the cell type most responsive to in vivo high-tidal-volume ventilation, demonstrating activation within just 1 min, followed by the alveolar epithelium. Alveolar macrophages were the slowest to respond, although they still demonstrated activation within 5 min. This order of activation was specific to VILI, since intratracheal lipopolysaccharide induced a very different pattern. These results suggest that alveolar macrophages may become activated via a secondary mechanism that occurs subsequent to activation of the parenchyma and that the lung cellular activation mechanism may be different between VILI and lipopolysaccharide. Our data also demonstrate that even very short periods of high stretch can promote inflammatory activation, and, importantly, this injury may be immediately manifested within the pulmonary vasculature.

mechanical ventilation is an integral tool in intensive care, but evidence shows that excessive tidal volumes can lead to ventilator-induced lung injury (VILI) (43) and increase mortality (2, 16). Patients with preexisting lung injury such as acute respiratory distress syndrome (ARDS) (30) are especially at risk since even low tidal volumes are enough to overstretch the reduced volume of aeratable lung (17, 46). Overdistension induces substantial inflammation (48, 55) and is ultimately thought to promote multiple-system organ failure (37, 44), the most common cause of death in ARDS patients (50). Furthermore, recent studies show that even patients with healthy lungs undergoing mechanical ventilation during surgery for relatively short lengths of time are susceptible to VILI. The use of ventilation strategies employing lower tidal volumes in the operating room has been shown to reduce the incidence of postoperative pulmonary complications and inflammation (41, 61). This stretch-induced “biotrauma” is therefore considered to be an important therapeutic target, but the risk of general immunosuppression means that “stretch-specific” processes must be identified.

It is therefore essential to understand how overstretch is sensed within the lung, and which cell types may be responsible for initiating the consequent inflammatory cascade, but as yet this remains unclear. Parenchymal (epithelial/endothelial) cells and resident leukocytes [alveolar macrophages (AMs)/lung-marginated monocytes] have all been demonstrated to be involved in VILI in some way, be it production of inflammatory mediators or changes in barrier function (12, 13, 22, 57). In vitro, many pulmonary cell types respond to mechanical deformation, including alveolar epithelial cells (AECs) (3, 33, 51, 59, 60), endothelial cells (20, 25), and AMs (38). It is uncertain though, precisely how relevant the in vitro conditions are to in vivo ventilation; for example, it seems unlikely that AMs would be exposed to the same stretching forces as structural cells in vivo. The simplest paradigm to explain the initiation of ventilator-induced inflammation would incorporate an initial stretch “sensor” followed by amplification and propagation of the inflammatory response, although this remains speculation and there is little hard evidence to support such a theory. We have therefore tested the hypothesis that, during high-stretch mechanical ventilation in vivo, AECs play the role of initial sensor and thus display activation of inflammatory pathways earlier than AMs, which we suggest would be the likely amplifiers of inflammation.

To explore this, we evaluated cellular inflammatory pathway activation in terms of phosphorylation of the mitogen-activated protein (MAP) kinases p38 and ERK1/2, p38’s immediate downstream substrate MAPK-activated protein kinase 2 (MK2), and the transcription factor NF-κB, during the first few minutes of VILI. p38 responds to a range of cell stresses and through activation of MK2 promotes the translation of proinflammatory mediators (32), whereas ERK1/2 is commonly associated with responses such as cell proliferation and differentiation (26). NF-κB is present in the cytoplasm of quiescent cells and upon phosphorylation rapidly translocates into the nucleus where it regulates many inflammatory genes (28). Activation of these markers has been frequently detected following high-tidal-volume ventilation in vivo (9, 29, 34, 49), and their inhibition attenuates aspects of VILI, including cytokine release (1, 8, 19, 20, 33, 42). However, these previous studies have used techniques such as Western blotting and immunohistochemistry, which are either not truly quantitative or do not allow localization of cellular activation. Furthermore, the earliest time point previously used to investigate in vivo MAP kinase activation is 30 min (49), which cannot be considered as related to the initiation of VILI. To circumvent these issues we developed a flow cytometry-based method to quantify MAP kinase and NF-κB phosphorylation in whole lungs. This methodology has the combined advantages of high detection sensitivity and the ability to localize activation to specific cell types following in vivo interventions.

The current study examined the kinetic profile of individual cell types within the first 15 min of exposure to high-tidal-volume ventilation in vivo and found that the pattern of inflammatory pathway activation was specific to VILI, compared with intratracheal lipopolysaccharide (LPS). Surprisingly, our data suggest that pulmonary endothelial cells initiate inflammatory signaling pathways at least as fast as AECs, and may be the most sensitive cell type to stretch of those tested, indicating that VILI directly induces pulmonary vascular inflammation from the start of its mechanotransduction process. AMs appeared to respond the slowest (although still within just 5 min), suggesting they may be activated by a secondary mechanism, albeit an extremely rapid one.

## METHODS

### 

#### In vivo models.

Protocols were approved by the UK Home Office in accordance with the Animals (Scientific Procedures) Act 1986, UK. A total of 96 male C57BL/6 mice (Charles River, Margate, UK) aged 10.9 ± 3.1 wk (mean ± SD), weighing 26.8 ± 3.0 g, were anesthetized by intraperitoneal injection of ketamine (90 mg/kg) and xylazine (10 mg/kg) before use as controls or exposure to either intratracheal LPS or VILI.

For LPS experiments, mice (*n* = 16) were exposed to intratracheal LPS for either 1, 5, or 15 min. For experiments lasting 5 or 15 min, 20 μg “Ultrapure” LPS (*Escherichia coli* O111:B4; InVivoGen, San Diego, CA) in a final volume of 50 μl saline was instilled intratracheally via a fine catheter briefly passed through the vocal chords, which were visualized using a microscope and external light source (36, 56). Animals were maintained spontaneously breathing under anesthesia and kept warm for the duration of the experiment. One minute before termination of the experiments, an endotracheal tube was inserted via tracheostomy to facilitate timely instillation of inhibitors as described below. To ensure accurate timing, in LPS experiments lasting only 1 min, the endotracheal tube was inserted first, before LPS (at the same dose and volume as above) was administered by passing the fine catheter through the endotracheal tube. Untreated mice (*n* = 11) were used as controls, since pilot experiments showed that administration of saline vehicle had no effect on cellular activation markers.

For VILI experiments, mice (*n* = 69) were connected to a custom-made ventilator through an endotracheal tube inserted via tracheostomy (55). Animals were initially ventilated with low-stretch settings: tidal volume (V_T_) 7–8 ml/kg, 120 breaths/min, and positive end-expiratory pressure (PEEP) of 3 cmH_2_O, using 100% oxygen. Two recruitment maneuvers were performed (sustained inflation of 35 cmH_2_O for 5 s) to standardize lung volume history before starting specific ventilation strategies. No further recruitments were performed during the timed ventilation exposure periods (since the longest of these was only 15 min). Mice were then randomly allocated to receive either injurious ventilation or to remain on the low-stretch settings. Initial experiments (26 mice) explored high-stretch ventilation (V_T_ 40 ml/kg_,_ 80 breaths/min, 3 cmH_2_O PEEP using O_2_ + 4% CO_2_) for 5 or 15 min, with 5 min of low stretch acting as controls. Subsequent experiments (14 mice) were carried out for 1 min with both high- and low-stretch settings. In a separate set of experiments (21 mice) lasting 5 min, ventilation with low stretch was compared with an intermediate stretch strategy (30 ml/kg, 80 breaths/min, 3 cmH_2_O PEEP using O_2_ + 4% CO_2_).

All experiments were terminated by exsanguination followed by immediate instillation of 500 μl cell-permeant phosphatase inhibitor cocktail (Calbiochem, Darmstadt, Germany) into the lungs via the endotracheal tube, to limit deterioration of the phosphoproteins. Lungs were rapidly removed within 2 min of termination and mechanically disrupted in warm (37°C) fixation/permeabilization buffer (Cytofix/Cytoperm; BD Biosciences, Oxford, UK) using a GentleMACS dissociator (Miltenyi Biotec, Surrey, UK). Samples were then incubated at 37°C for a further 10 min, following which fixation was stopped by addition of ice-cold permeabilizing buffer (phosphate-buffered saline, 0.2% saponin, 2% fetal calf serum, and 0.1% sodium azide). Samples were filtered through 40-μm sieves and finally washed/resuspended in permeabilizing buffer (to ensure access of antibodies to the cytoplasmic phosphoproteins) to yield a fixed single cell suspension of the whole lung as previously described (31).

#### Phosphoflow cytometry.

Lung cell suspensions were stained for 30 min at room temperature with fluorophore-conjugated antimouse antibodies to identify pulmonary cell populations. Epithelial cell populations were identified using antibodies against CD45-PerCP (clone 30-F11; BioLegend, San Diego, CA), CD31-FITC (MEC 13.3; BioLegend), EpCAM-PE (G8.8; eBioscience, San Diego, CA), and T1-α-PeCy7 (8.1.1; BioLegend) (15, 35). In addition, type I and type II AECs were further differentiated based on positive or negative staining for CD54 (ICAM-I)-FITC (3E2; BD Biosciences, San Jose, CA). Endothelial cells were identified using antibodies against CD45-PerCP and CD31-FITC (4) and were also confirmed as staining positive for CD105-PE (MJ7/18; eBioscience) and negative for the platelet marker CD41-FITC (MWReg,30; Serotec, Oxford, UK). AMs were identified using antibodies against CD45-PerCP, CD11b-PE-CF594 (M1/70; BD Horizon), F4/80-FITC (BM8; BioLegend), and CD11c-Ax780 (N418; eBioscience) (5).

With the use of a previously published technique (24, 31), lung cell suspensions were also stained with AF-647-conjugated antibodies against intracellular phospho-p38 (Thr^180/182^; clone 28B10), phospho-MK2 (Thr^334^; 27B7), phospho-ERK1/2 (Thr^202^/Tyr^204^; D13.14.4E), and phospho-NF-κB p65 (Ser^536^; 93H1) (Cell Signaling, Danvers, MA). To validate the findings, in a separate set of experiments eight animals were ventilated with the high-stretch or low-stretch settings for 5 min, and levels of both phosphorylated and total (phosphorylated plus nonphosphorylated) ERK1/2 (137F5; Cell Signaling) were determined within the same lungs. Samples were analyzed with a CyAn ADP flow cytometer (Beckman Coulter, High Wycombe, UK) and FlowJo software version 10 (TreeStar, Ashland, OR). Signals were determined as geometric mean of fluorescence intensity (gMFI).

#### Statistical analysis.

Shapiro-Wilk normality and Levene's homogeneity of variance tests were conducted on all data. Where possible, nonparametric data were normalized using square root transformation. Comparisons between two datasets were performed using *t*-tests or Mann Whitney *U*-tests. Comparisons between three or more datasets were performed using ANOVA with Tukey's honest significant difference, Welch ANOVA with Games-Howell for parametric data with unequal variance, or Kruskal Wallis with Dunn's test for nonparametric data. Statistical significance was defined as *P* < 0.05. Data were analyzed and graphed using IBM SPSS (version 20) and Prism software (version 6).

## RESULTS

### 

#### Intratracheal LPS model.

**Fig. 1.**
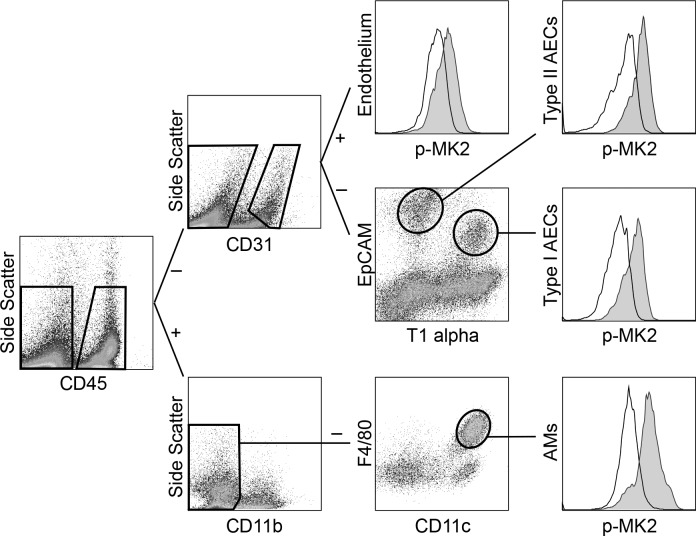
Gating strategies for identifying specific cell types from a whole lung single cell suspension using flow cytometry. Alveolar macrophages were identified as CD45^+^, CD11b^−^, F4/80^+^, and CD11c^+^. Endothelial cells were identified as CD45^−^, CD31^+^ and were also confirmed as staining positive for CD105 and negative for the platelet marker CD41. Type I alveolar epithelial cells (AECs) were identified as CD45^−^, CD31^−^, EpCAM^+^, and T1-α^+^ and type II AECs as CD45^−^, CD31^−^, EpCAM^+^, and T1-α^−^. In addition, type I and type II AECs were further differentiated based on positive or negative staining for CD54 (ICAM-I). Representative histograms display the increase in geometric mean fluorescence intensity (gMFI) of phospho-MAPK-activated protein kinase 2 (MK2) after 15 min of exposure to intratracheal lipopolysaccharide (LPS) (gray) vs. untreated (white).

**Fig. 2.**
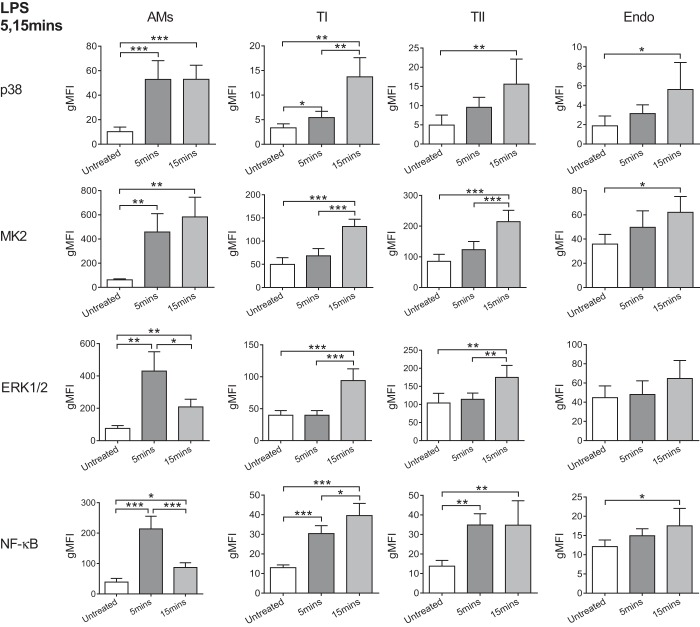
Phosphorylation levels (gMFI) of the activation markers for all cell types after exposure to intratracheal LPS for 5 or 15 min compared with untreated controls. T1, type I AECs; TII, type II AECs; Endo, endothelium. Alveolar macrophages (AMs) and epithelial cells displayed responses to LPS within just 5 min; *n* = 5 experiments. *P < 0.05, **P < 0.01, and ***P < 0.001. Data were analyzed by ANOVA with Tukey's honest significant difference (HSD), or Welch ANOVA with Games-Howell tests for parametric data with unequal variance. Data are displayed as means ± SD.

Initial experiments (for both the LPS and VILI models) were carried out over 5- and 15-min periods. Figure 1 shows the gating strategies used to identify the different pulmonary cell types, and for each a representative overlay of histograms shows the increase in phospho-MK2 after 15 min of LPS compared with an untreated control. Intratracheal LPS induced significant activation in each of the cell types studied in this experiment, with the clearest response exhibited by the AMs (Fig. 2). In the AMs, a more than fivefold increase in the mean fluorescence intensity (gMFI) of phospho-p38 was detected at both 5 and 15 min after LPS instillation. MK2 also displayed sustained activation in response to LPS, with a seven- to ninefold increase in phosphorylation after 5 and 15 min. In contrast, ERK1/2 and NF-κB demonstrated a more transient activation that peaked at 5 min. Phosphorylation of both of these markers decreased considerably by 15 min, although they were still significantly increased compared with their respective baseline levels.

Compared with the AMs, the type I and II AECs both showed clear activation of NF-κB within 5 min, although most of the MAP kinases only reached significant levels of activation after 15 min. The endothelial response was less apparent than that of epithelial cells (Fig. 2), with no significant activation of any marker at 5 min. NF-κB took 15 min to become significantly activated and was accompanied by activation of p38 and MK2 (1.5-fold increase in signal), although ERK1/2 activation failed to reach significance at all.

#### VILI model.

**Fig. 3.**
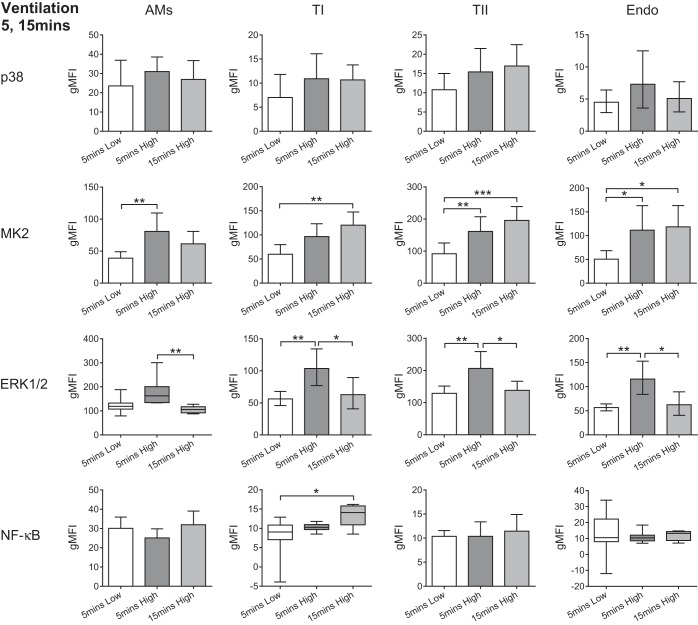
Phosphorylation levels (gMFI) of the activation markers for all cell types after exposure to 5 or 15 min of high-stretch ventilation compared with 5 min of low stretch. The parenchymal cells appeared more responsive to stretch than the AMs; *n* = 5–8. *P < 0.05, **P < 0.01, and ***P < 0.001. Data were analyzed by ANOVA with Tukey's HSD and are displayed as means ± SD, or geometric mean with 95% confidence intervals (lower, upper bounds) for transformed data. Nonparametric data were analyzed by Kruskal-Wallis with Dunns and are displayed as box-whisker plots with error bars extending to minimum and maximum values.

Compared with LPS, high-stretch ventilation induced a markedly different pattern of cellular activation, with all the cell types demonstrating inflammatory signaling pathway activation within just 5 min (Fig. 3). The AMs displayed significantly increased phosphorylation of MK2 at 5 min, albeit transiently. The type I AECs displayed transient ERK1/2 activation that peaked at 5 min, whereas MK2 displayed a trend for increased phosphorylation at 5 min that became significant at 15 min. The type II AECs exhibited similar responses to the type I AECs, with transient ERK1/2 activation peaking at 5 min and increased MK2 phosphorylation at both time points. As with all the other cell types, the endothelial cells displayed a transient increase in ERK1/2 phosphorylation that peaked at 5 min. MK2 was also significantly activated by 5 min and demonstrated sustained activation at 15 min. Interestingly, unlike the situation following LPS stimulation, there was very little evidence of NF-κB activation in response to high stretch, with only type I AECs showing increased NF-κB phosphorylation after 15 min of stretch (Fig. 3).

**Fig. 4.**
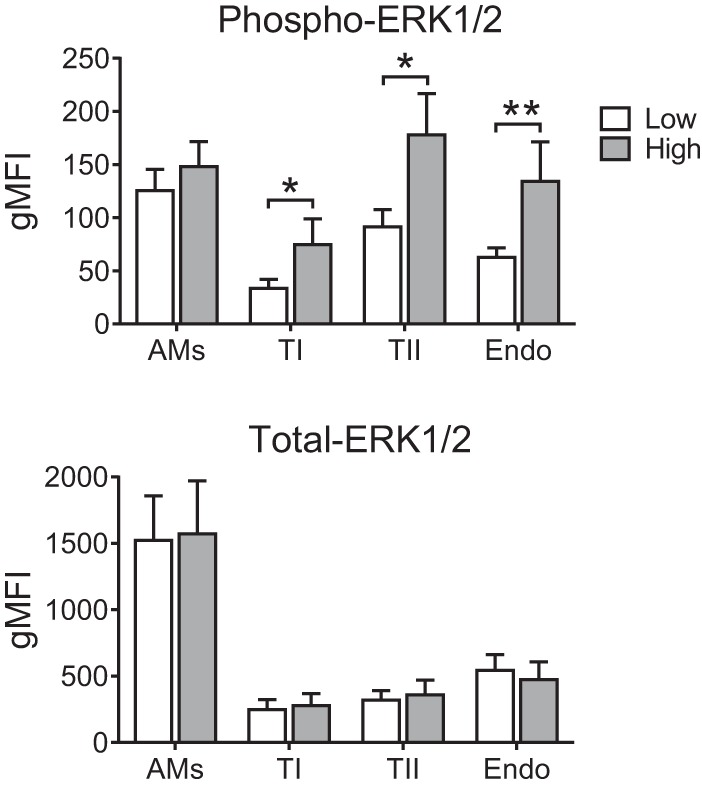
Levels of phosphorylated and total ERK1/2 protein within lungs subjected to 5 min of low- or high-stretch ventilation. Consistent with findings from Fig. 3, levels of phospho-ERK1/2 clearly increased with high-stretch ventilation, whereas levels of total ERK1/2 within the same lungs were unaltered; *n* = 4. *P < 0.05 and **P < 0.01. Data were analyzed by *t*-test and are displayed as means ± SD.

To further validate this discovery of widespread MAP kinase phosphorylation in response to stretch, in particular given that it was more subtle than LPS-induced activation, we carried out a separate set of experiments to quantify the levels of both phosphorylated and total ERK1/2 protein within the same lungs after 5 min of high- or low-stretch ventilation. ERK1/2 was chosen for these experiments since it was consistently upregulated at 5 min within most cell types, and there is no commercially available, flow cytometry validated total MK2 antibody. These experiments once again demonstrated significantly increased phosphorylation of ERK1/2 in response to high-stretch ventilation, whereas the total levels of ERK1/2 protein were not altered (Fig. 4).

#### Parenchymal cells are the initial “inflammatory responders.”

Because all cell types examined were found to demonstrate activation following as little as 5 min of high-stretch mechanical ventilation, it was not possible to draw any conclusions regarding which cell types may be the initiating cells, responsible for starting the inflammatory cascade. To address this, we carried out two further sets of experiments: *1*) an investigation of an even earlier time point for both LPS and VILI experiments (1 min), and *2*) an investigation of lower tidal volumes for the VILI model.

**Fig. 5.**
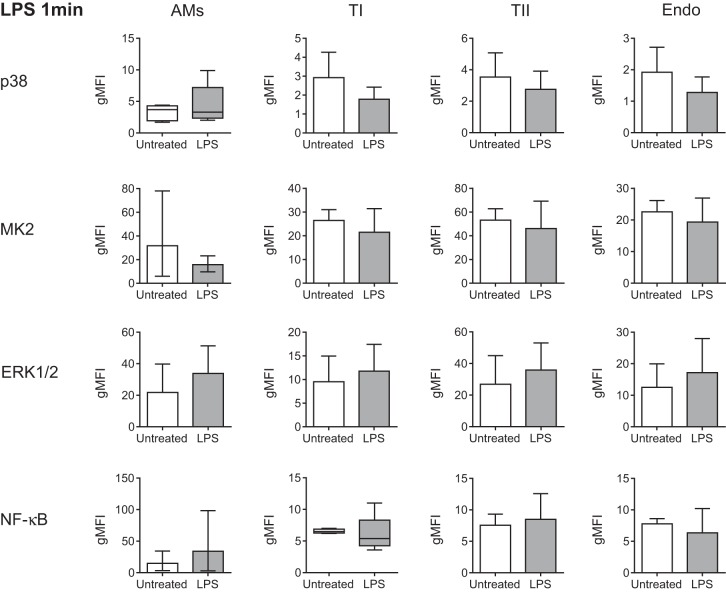
Phosphorylation levels (gMFI) of the activation markers for all cell types after exposure to intratracheal LPS for 1 min compared with untreated controls. No significant signs of activation could be detected after 1 min of LPS; *n* = 4–6. Data were analyzed by *t*-test and are displayed as means ± SD, or geometric mean with 95% confidence intervals (lower, upper bounds) for transformed data. Nonparametric data were analyzed by Mann Whitney *U* and are displayed as box-whisker plots with error bars extending to minimum and maximum values.

**Fig. 6.**
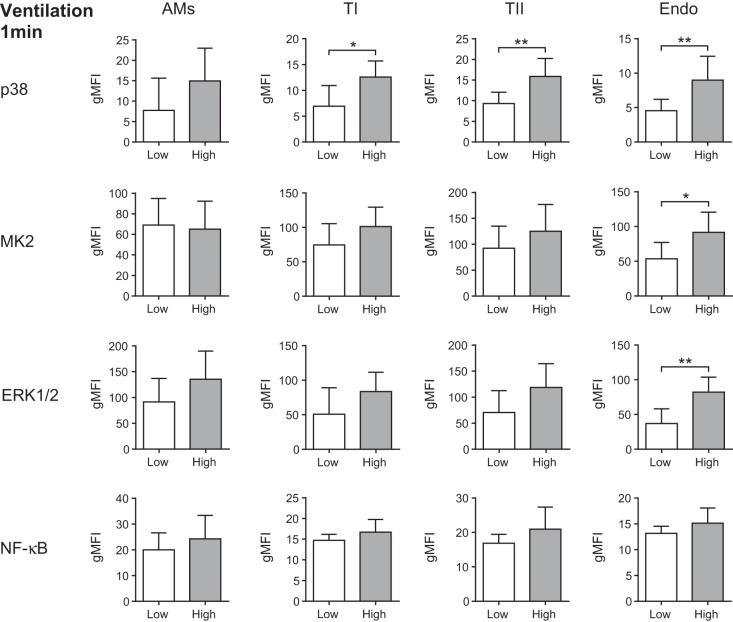
Phosphorylation levels (gMFI) of the activation markers for all cell types after exposure to 1 min of low- or high-stretch ventilation. The endothelial cells displayed activation of the most markers after just 1 min of high-stretch ventilation; *n* = 5–7. *P < 0.05 and **P < 0.01. Data were analyzed by *t*-test and are displayed as means ± SD.

**Fig. 7.**
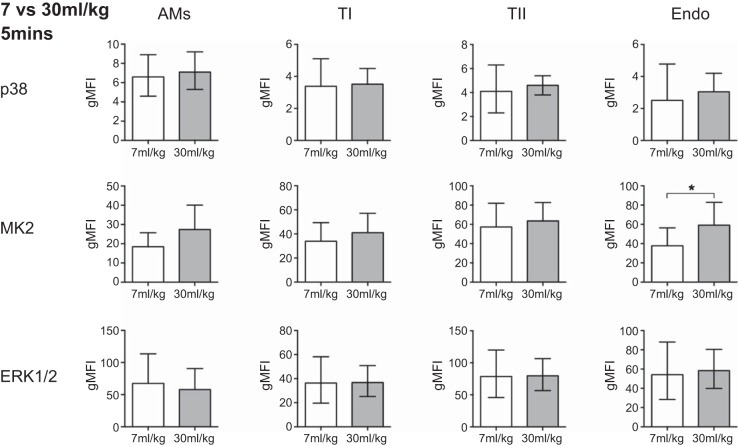
Phosphorylation levels (gMFI) of the activation markers for all cell types after exposure to 5 min of ventilation at 30 ml/kg compared with 5 min of low-stretch ventilation. Only the endothelial cells demonstrated sensitivity to the intermediate tidal volume of 30 ml/kg; *n* = 7–11. *P < 0.05. Data were analyzed by *t*-test and are displayed as means ± SD, or geometric mean with 95% confidence intervals (lower, upper bounds) for transformed data.

Despite clear activation of AMs after 5 min of exposure to intratracheal LPS, there was no significant activation at 1 min (Fig. 5). The type I and II AECs, which both demonstrated NF-κB phosphorylation after 5 min of LPS, showed no evidence of activation after 1 min, and neither (unsurprisingly) did the endothelium (Fig. 5). In contrast, just 1 min of high-stretch ventilation induced significant cellular activation within the lung (Fig. 6). Both type I and II AECs displayed significantly increased p38 activation, while, somewhat surprisingly, the endothelial cells demonstrated the clearest signs of significant activation, with p38, MK2, and ERK1/2 all showing approximately twofold increases in phosphorylation (Fig. 6). In contrast, AMs showed no significant activation, suggesting that they were responding less rapidly than other cell types (Fig. 6). Finally, experiments conducted at a lower tidal volume of 30 ml/kg for 5 min only induced significant MAP kinase (MK2) activation in endothelial cells (Fig. 7), further indicating that they may be the most sensitive to stretch during ventilation.

## DISCUSSION

This study was designed primarily to investigate which pulmonary cell types initiate the inflammatory sequelae following overdistension during high-tidal-volume ventilation. Until now, research has focused on the downstream consequences of VILI, such as cytokine release, but very little is known about the in vivo mechanotransduction and signal propagation processes underlying the initiation of inflammation. We have investigated intracellular inflammatory signaling events within the first 15 min after initiating high-tidal-volume ventilation in vivo and compared this with intratracheal LPS administration to see whether we could identify a stretch-specific pattern. We used a flow cytometric technique previously established within our group (31) to quantitatively assess the phosphorylation status of intracellular MAP kinases, which provides the advantages of measuring an upstream signal transduction event, with cell-specific localization of the signal.

This novel approach has revealed differential responses of pulmonary cells in vivo to a bacterial and mechanical insult. The bacterial endotoxin LPS induced activation of multiple inflammatory pathways just 5 min after intratracheal instillation into the lungs. This rapidity of response is consistent with previous observations of MK2 phosphorylation in the whole lung (40), but our data allow us to clarify the cells responsible. The data show a distinct pattern of activation within cell types, with AMs showing activation of MAP kinases and NF-κB at 5 min, whereas epithelial cells primarily showed activation of NF-κB at this early time point. These data are mainly consistent with previous in vitro studies indicating that LPS induces cytokine secretion in macrophages through activation of ERK1/2 and p38/MK2 pathways, whereas epithelial cell cytokine production is not mediated through these MAP kinases (47). Interestingly, these in vitro studies showed no ERK1/2 or p38 activation in epithelial cells for up to 4 h following LPS, whereas we found phosphorylation of these within 15 min. This is likely to suggest that this in vivo response is indicative of a secondary activation via a paracrine mediator, for example, TNF. In contrast to AMs and epithelial cells, endothelium showed no signs of activation until 15 min. The fact that this activation included not only NF-κB but also MAP kinases, along with the delay in response, may suggest that this is also secondary mediator-induced activation.

Compared with LPS, injurious mechanical ventilation induced an entirely different sequence of events. Each of the individual cell types studied showed signs of significant inflammatory pathway activation within 5 min, although interestingly this did not include NF-κB, which was the most consistently activated marker 5 min after LPS. The rapidity of this response to ventilation led us to explore even earlier time points, finding that the endothelial cells displayed activation of all MAP kinase markers after 1 min of high stretch, while the AECs displayed some signs (increased phosphorylation of p38) of becoming activated at this time point. AMs, however, displayed no significant increases in any marker at 1 min.

Taken together these data indicate that parenchymal cells are likely to be the cells within the lungs that initiate the inflammatory response to stretch, followed by communication to other cell types, which can then propagate the cascade. Within the alveolar space, this would seemingly take the form of stretched epithelial cells sending a signal to AMs. Many in vivo studies have localized MAP kinase and NF-κB activation to the alveolar epithelium using immunohistochemistry, although only after longer periods of high-tidal-volume ventilation (1, 29, 49) than used presently. One previous in vivo study indicated that AMs could be activated by soluble signals contained within bronchoalveolar lavage fluid taken from animals ventilated with high stretch for 20 min (13), although it was not clarified whether said mediator(s) actually came from epithelial cells within that study. The nature of this signal is therefore not yet clear but according to our data is limited to mechanisms that could stimulate macrophage responses within 5 min. Possible options include *1*) soluble mediators such as prostaglandins, which have been shown to be rapidly released from AECs stretched in vitro within a 10-min time frame (8), *2*) direct communication between epithelial cells and macrophages through gap junction channels (54), or *3*) indirect sensing of epithelial cell deformation mediated through adhesive contacts between macrophages and the epithelium.

Intriguingly, our data indicate that endothelial cells were potentially not only the first cell type to activate inflammatory pathways during high stretch but also the most sensitive to lower levels of stretch. Indeed, Dreyfuss and Saumon showed nearly 20 years ago that just 5 min of high-pressure ventilation induced endothelial cell blebbing (11), and increased p38 and ERK1/2 activation has been detected after 5 min in pulmonary endothelial cells stretched in vitro (1, 20, 23). To the best of our knowledge, our study is the first report of such rapid endothelial MAP kinase activation following in vivo ventilation. One of the fundamental functions of endothelial cells is to control vascular tone in response to mechanical forces applied onto them, i.e., shear stress by blood flow and circumferential stretch force generated by vascular transmural pressure. During high-tidal-volume ventilation, both the shear stress and circumferential stretch would likely decrease because of somewhat reduced pulmonary flow and capillary compression (in a direction perpendicular to the alveolar wall) by positive alveolar pressure. However, at the same time, because of the physical nature of the capillary network as a mesh surrounding alveoli, firmly attached to the alveolar wall via the basement membrane, lung inflation would produce significant “longitudinal” stretch of the capillary wall, as proposed by West as part of his theory of capillary stress failure (14, 53). The rapidity of the endothelial response in our experiments supports the likelihood of such longitudinal capillary deformation, indicating that these endothelial cells are indeed responding directly to stretch rather than a released secondary mediator. Our results would seem to be consistent with the hypothesis that pulmonary capillary endothelial cells may be by nature more responsive than AECs to such longitudinal stretch of the alveolar wall during lung inflation and suggest a potentially central role for endothelial cells in initiating VILI.

These data may have a further important implication in respect to the systemic propagation of inflammation. Lung-derived mediators produced during VILI have been proposed to play significant roles in causing injury to nonpulmonary organs, but, while studies have attempted to identify the nature of such mediators (21), the cell source has received relatively little attention. The “decompartmentalization” hypothesis suggests a leak of mediators from the alveolar space to the circulation, but the very rapid activation of inflammatory pathways in endothelial cells in our study suggests that other mechanisms are likely to be involved. We have previously shown that lung-marginated monocytes play an important role in VILI in vivo (57) and in systemic cytokine release during in situ ventilation (52). Taken together, these data could suggest that, in a process analogous to that proposed in the alveolar space, endothelial cells initially respond to deformation and then pass signals to leukocytes marginated within the capillaries to amplify the inflammatory signal systemically.

The tidal volumes used in this study are much higher than those used clinically. However, they do reflect the degree of stretch experienced by heterogeneously injured human lungs (17, 46, 58), which are inherently less compliant than those of mice (45). Furthermore, tidal volumes clinically relevant to human ARDS are not necessarily always ideal for experimental purposes in mice. Although many examples exist of mouse models demonstrating the development of deteriorated lung function with more “clinically consistent” tidal volumes (18, 27), it is often not clear to what extent stretch, per se, rather than other confounding factors, such as atelectasis or a preceding lung insult, is the primary cause of injury (39, 58). Indeed, we have previously demonstrated that tidal volumes up to 20 ml/kg are unlikely to induce substantial stretch within the lungs of healthy mice (58). The current study was designed to address the cellular in vivo responses to stretch specifically, and therefore the most appropriate model was deemed to be one in which stretch would predominate. Although we did not determine blood pressure or blood gases directly in this study (to minimize surgical instrumentation and potential extra inflammatory insult), we routinely use the same ventilator settings in our mouse VILI experiments and do not observe any deterioration in blood pressure or gases until well after the 15-min time frame of the current study (10, 55, 58). Furthermore, because there was virtually no NF-κB response to ventilation, the possibility that activation was the result of contamination by LPS appears minimal. Overall, we therefore believe that it is highly likely that the activation measured in the current study is a direct consequence of lung overstretch.

Finally, it is important to clarify that in the current study we were attempting specifically to explore the initiation of inflammatory cascades within the lungs, rather than identify which cells may be “responsive” per se to stretch. For example, one large breath is capable of mobilizing intracellular calcium and promoting surfactant secretion from epithelia (60). However, such responses do not necessarily have immediate relevance to the way in which inflammatory cascades and intercellular communication begin. It remains possible that inflammatory pathways other than the ones we have measured are upregulated; however, the markers used are involved in three separate signaling pathways that promote inflammatory responses to myriad stress stimuli. Many of the markers displayed transient activation in response to both LPS and high-stretch ventilation, a pattern that seems to be characteristic of the MAP kinases and NF-κB. Indeed, many in vitro studies have also detected transient patterns of activation in response to both stretch and LPS in a variety of cell types (1, 6, 7, 40). Exploring the mechanisms behind this transience was outside the scope of the current study but may include processes such as translocation into the nucleus for NF-κB (a compartment inaccessible to the antibodies under the current protocols) and enhanced activity of phosphatases as part of an autoregulation process. Regardless, p38, ERK1/2, and NF-κB have been frequently implicated in inflammatory signaling initiated by VILI, and their inhibition has been shown to attenuate symptoms of VILI, including cytokine release (19, 20, 33), suggesting that even very transient activation is sufficient to induce an inflammatory cascade.

In summary, our data demonstrate a substantially differing cellular activation profile between mechanical stretch and intratracheal LPS. By studying the immediate responses to challenge, we have identified likely “initial responders,” and the cells that become activated later, potentially in response to secondary signals. We propose that blocking lines of communication between cell types or amplification of signals has the potential to be a safer, more targeted approach to patient treatment for VILI. Our data suggest that the nature of mediators/lines of communication is likely to be different between mechanical ventilation and bacterial challenge. The protocols developed here will allow us to explore in detail the processes responsible, and when these are identified it may be possible in the future to attenuate the ventilation-induced exacerbation of local and systemic inflammation while retaining immune responses to infection intact.

## GRANTS

This work was supported by grants from the British Journal of Anaesthesia/Royal College of Anaesthetists and the Wellcome Trust (no. 081208).

## DISCLOSURES

No conflicts of interest, financial or otherwise are declared by the authors.

## AUTHOR CONTRIBUTIONS

Author contributions: S.J.W., A.A.C.W., and P.H. performed experiments; S.J.W., A.A.C.W., K.P.O., P.H., M.T., and M.R.W. analyzed data; S.J.W., A.A.C.W., K.P.O., P.H., M.T., and M.R.W. interpreted results of experiments; S.J.W. prepared figures; S.J.W. drafted manuscript; S.J.W., M.T., and M.R.W. edited and revised manuscript; S.J.W., A.A.C.W., K.P.O., P.H., M.T., and M.R.W. approved final version of manuscript; A.A.C.W., K.P.O., M.T., and M.R.W. conception and design of research.
